# Calcific Uremic Arteriolopathy (CUA) Among Malaysian Dialysis Patients: Clinical Characteristics, Treatment, and Outcomes

**DOI:** 10.7759/cureus.73711

**Published:** 2024-11-14

**Authors:** Yee Wan Lee, Min Hui Tan, Sunita Bavanandan, Boon Cheak Bee, Christopher Thiam Seong Lim, Rozita Mohd, Yew Foong Liew, Chek Loong Loh, Wen Jiun Liu, Fariz Safhan Mohamad Nor, Zaiha Harun, Korina Rahmat, Clare Hui Hong Tan, Koh Wei Wong, Soo Kun Lim

**Affiliations:** 1 Department of Nephrology, Universiti Malaya Medical Centre, Kuala Lumpur, MYS; 2 Department of Nephrology, Hospital Kuala Lumpur, Kuala Lumpur, MYS; 3 Department of Nephrology, Hospital Selayang, Selayang, MYS; 4 Department of Medicine, Universiti Putra Malaysia, Serdang, MYS; 5 Department of Medicine, Faculty of Medicine, Universiti Kebangsaan Malaysia, Kuala Lumpur, MYS; 6 Department of Nephrology, Hospital Pulau Pinang, Penang, MYS; 7 Department of Nephrology, Hospital Raja Permaisuri Bainun, Ipoh, MYS; 8 Department of Nephrology, Hospital Sultanah Aminah, Johor Bahru, MYS; 9 Department of Nephrology, Hospital Tengku Ampuan Afzan, Kuantan, MYS; 10 Department of Nephrology, Hospital Sultanah Nur Zahirah, Kuala Terengganu, MYS; 11 Department of Nephrology, Hospital Melaka, Melaka, MYS; 12 Department of Nephrology, Hospital Umum Sarawak, Kuching, MYS; 13 Department of Nephrology, Hospital Queen Elizabeth, Kota Kinabalu, MYS

**Keywords:** calcific uremic arteriolopathy, ckd-mbd, dialysis, end-stage renal disease (esrd), mineral bone diseases

## Abstract

Background

Calcific uremic arteriolopathy (CUA) is a rare but debilitating disease affecting patients with kidney disease. Reported risk factors of CUA in the literature include female sex, obesity, diabetes mellitus, and vitamin K antagonists' (VKAs) usage. CUA prevalence in Malaysia is unknown and has not been reported before.

Methods

A multicenter observational study was conducted in 13 centers all over Malaysia to study the clinical characteristics, treatment, and outcomes of CUA. The data of patients confirmed with CUA between January 1, 2016, and December 31, 2021, was collected from medical records by each center’s nephrologists.

Results

Out of 33 confirmed CUA cases, 69.7% were females, and 66.7% were Malay with a mean age of 47.33 ± 13.80 years old. The mean BMI was 25.66 ± 9.77 kg/m^2^, and 18.2% were classified as obese (BMI > 30). Two-thirds of the patients were on hemodialysis (HD), and the mean dialysis vintage was 6.2 ± 4.11 years. A majority (87.9%) have hypertension, 33.3% are diabetic, and 27.3% have coronary artery disease. Only 15.2% of the patients were on warfarin at the time of diagnosis. A total of 78.8% of patients were taking calcium-based phosphate binders during diagnosis. Investigation results showed calcium, 2.44 ± 0.29 mmol/L; phosphate, 2.18 ± 0.67 mmol/L; CaXPO4 = 5.41 ± 1.90; and parathyroid hormone, 181.14 ± 153.23 pmol/L. About half (54.5%) had skin biopsy confirmation done. Distribution of lesions was 57.6% peripheral and 30.3% central. For treatment of CUA, there were 57.6% usage of non-calcium-based phosphate binders, 48.5% cinacalcet, 30.3% sodium thiosulphate, and 33.3% had parathyroidectomy. Half (54.5%) of our CUA patients died within three months from diagnosis. The mean time from diagnosis to mortality was 4.12 ± 5.59 months. A majority (45.5%) died from septicemia caused by infections. Interestingly, there were a few rare presentations of CUA such as pulmonary calciphylaxis, heart and lung calcifications, liver and spleen calcifications, and genital lesions. One patient had resistant CUA and was given a trial of lipid apheresis for 10 sessions.

Conclusion

This is the first and largest multicenter study looking into the characteristics, treatment, and outcome of CUA in Malaysia. Majority of patients in Malaysia undergo HD as kidney replacement therapy; hence, our results correlate with this. The incidence of CUA was estimated to be 6.6 per 10,000 dialysis patients in this study and the mortality rate is very high. This is consistent with worldwide data which reported mortality as high as 60%.

## Introduction

This article was previously presented as a poster at the International Society of Nephrology (ISN) World Congress of Nephrology (WCN) 2023 on March 30 to April 2, 2023, in Bangkok, Thailand.

Calciphylaxis is a rare but debilitating condition typically presented with painful skin lesions which are caused by calcification of arterioles and capillaries in the dermis and subcutaneous adipose tissues [1]. The term calcific uremic arteriolopathy (CUA) is preferred to describe this condition in patients with end-stage kidney disease (ESKD). Calciphylaxis may occur in kidney transplant recipients and non-ESKD patients. 

CUA has been rarely reported worldwide. A report from the United States estimates an annual incidence of 35 cases per 10,000 hemodialysis (HD) patients [2]. Meanwhile, other reports show four per 10,000 in Germany [3] and less than one per 10,000 in Japan [4]. A cross-sectional study of 242 HD patients in Hawaii reported a prevalence of 4% [5].

Pathogenesis of CUA is poorly understood. Factors that are presumed to be involved are due to hyperparathyroidism, abnormalities of calcium and phosphate metabolism, deficiencies of inhibitors of vascular calcifications such as matrix Gla protein and Fetuin A [1]. Patients typically presents with painful, nonhealing wounds which progresses to sepsis and death.

Reported risk factors of CUA in the literature are female sex, obesity, diabetes mellitus, use of vitamin K antagonists (VKAs), and ESKD [1]. A case control study of 89 French patients shows that CUA mainly involved obese patients on VKA [6]. McCarthy et al. reported 80% females, 70% obese, and 60% usage of VKA patients in his study of 101 calciphylaxis patients [7].

The overall prognosis of this condition is poor, with a reported one-year mortality rate of 45%-80% [2,7-8]. There is currently no approved treatment for CUA. Patients are usually treated with multidisciplinary approach which include wound care, pain control, and optimized nutrition and dialysis and palliative care.

There has been no reported literature on the epidemiology, risk factors, treatment, and outcome of CUA in Malaysia. Therefore, we conduct this multicenter study to evaluate the clinical characteristics, risk factors, treatment, and outcome of CUA among Malaysian dialysis patients. The availability of a registry of CUA patients may prompt further research among experts and clinicians in this field of vascular calcification.

## Materials and methods

We conducted a retrospective multicenter observational study on the epidemiology, risk factors, treatment, and outcomes of CUA in Malaysia. There was a total of 13 centers all over Malaysia involved in this study. We retrospectively reviewed data of patients confirmed to have CUA between January 1, 2016, and December 31, 2021, in all the centers involved. Data collection was performed by tracing medical records by the nephrologist in each center who is caring for the patients. This study protocol was reviewed and approved by the University of Malaya Medical Centre Medical Research Ethics Committee, with approval number 2021922-10603. The requirement for informed consent was waived due to the retrospective nature of the study.

Inclusion criteria included patients aged 18 years or older and patients on HD or peritoneal dialysis (PD) who are at least three months after dialysis initiation with a confirmed diagnosis of CUA by the Hayashi criteria [4]. In 2013, Hayashi proposed a diagnostic criterion for CUA which can be made when patients on chronic HD or estimated glomerular filtration rate (eGFR) below 15 ml/min/1.73m^2^ presented with more than two painful, nontreatable skin ulcers with concomitant painful purpura and localization of skin ulcers on the trunk, extremities, or penis with concomitant painful purpura [4].

If a skin biopsy was done, the typical histopathological findings showing necrosis and ulceration of the skin with calcification of the tunica media and internal elastic membrane of small- to medium-sized arterioles of dermis and subcutaneous fat can replace a clinical feature [4]. We excluded patients who were diagnosed with calciphylaxis but was not on any form of dialysis, kidney transplant patients, and skin lesions that are not CUA. For each CUA case, central lesions was defined as lesions at the chest, trunk, buttocks, genitals, lower extremities proximal to the knee joints, and upper extremities proximal to the elbow joints, while peripheral lesions was defined as lesions restricted to the lower extremities at and distal to the knee joints, upper extremities at and distal to the elbow joints, and the head [4].

Data collected includes demographic details, comorbidities, risk factors of CUA, treatment, and outcome of the patient. Date of onset of CUA was taken from the date of diagnosis when the typical skin lesions was first documented in the medical records. Dialysis blood parameters was taken from the worst values recorded in medical records within the six months from diagnosis of CUA. For the intact parathyroid hormone (iPTH) measurement, different centers were using different kits, so the results were normalized with the upper limit of the normal range for each individual laboratory. For laboratories that was using pg/ml, the value was converted to pmol/L by dividing by 9.4. Medications that patients were taking while diagnosed with CUA were recorded. Treatment methods, outcome, time to mortality, and cause of mortality were collected.

The frequency of categorical variables, mean ± standard deviation for normally distributed data, median, and interquartile range (IQR) for nonnormally distributed variables were reported. All analyses were performed using the IBM SPSS Statistics for Windows, Version 23 (Released 2015; IBM Corp., Armonk, New York, United States). Significance was set as p < 0.05.

## Results

A total of 33 patients were diagnosed with CUA during the study period. The mean age was 47.33 ± 13.80 years old. A majority (69.7%) of the patients were female and 66.7% were Malay. The mean BMI was 25.66 ± 9.77 kg/m^2^, and 18.2% were classified as obese with obesity defined as BMI > 30. Among our patients, 66.7% were on hemodialysis as the mode of renal replacement therapy, while the rest were PD. The mean dialysis vintage was 6.2 ± 4.11 years.

A majority (87.9%) of our patients had concomitant hypertension, 33.3% diabetes mellitus, 51.5% dyslipidemia, and 27.3% coronary artery disease. Only 15.2% of the patients were on warfarin at the time of diagnosis. A total of 78.8% of patients were taking calcium-based phosphate binders, and only 39.4% were on non-calcium-based phosphate binders during diagnosis. Relevant blood investigation results showed calcium, 2.44 ± 0.29 mmol/L; phosphate 2.18 ± 0.67 mmol/L; CaXPO4 = 5.41 ± 1.90; alkaline phosphatase, 364.47 ± 324.68 U/L; and parathyroid hormone, 181.14 ± 153.23 pmol/L.

About half (54.5%) of our patients had skin biopsy confirmation done. The distribution of lesions was 57.6% peripheral, 30.3% central, and 12.1% had both central and peripheral lesions. We recorded a few rare presentations of CUA which included pulmonary calciphylaxis, heart and lung calcifications, liver and spleen calcifications, and genital lesions.

For the management of CUA, 57.6% were changed to non-calcium-based phosphate binders, 48.5% was started on cinacalcet, 30.3% required the use of sodium thiosulphate, 33.3% underwent parathyroidectomy, and 33.3% required intensification of dialysis frequency. As all patients had typical lesions of CUA, 93.9% needed expert wound care, while 33.3% required surgical debridement. One patient had resistant CUA and was given a trial of lipid apheresis for 10 sessions.

Half (54.5%) of our CUA patients died within three months from diagnosis. The mean time from diagnosis to mortality was 4.12 ± 5.59 months. Majority (45.5%) died from septicemia caused by infections. Further details are as listed in Table 1.

**Table 1:** Clinical characteristics, treatment, and outcome of patients BMI: Body mass index; CUA: calcific uremic arteriolopathy; ESA: erythropoiesis-stimulating agents; HD: hemodialysis; PD: peritoneal dialysis; IV: intravenous

Total patients (n)	33
Total hospital/centers	13
Age (years)	47.33 ± 13.80
Gender	Male = 10 (30.3%)
	Female = 23 (69.7%)
Race	Malay = 22 (66.7%)
	Chinese = 9 (27.3%)
	Others = 2 (6.1%)
Weight (kg)	67.94 ± 22.67
BMI	25.66 ± 9.77
	Obese (BMI > 30) = 6 (18.2%)
Dialysis details	HD = 22 (66.7%)
	PD = 11 (33.3%)
	Dialysis vintage (years) = 6.2 ± 4.11
	kt/V (HD) = 1.28 ± 0.32
	kt/V (PD) = 1.73 ± 0.19
Comorbidities	Diabetes mellitus = 11 (33.3%)
	Hypertension = 29 (87.9%)
	Dyslipidemia = 17 (51.5%)
	Coronary artery disease = 9 (27.3%)
	Heart failure = 11 (33.3%)
	Stroke = 1 (3.0%)
	Connective tissue disease = 3 (9.1%)
	Cancer = 5 (15.2%)
	History of pathological fractures = 3 (9.1%)
	Smoking = 3 (9.1%)
Medications taken during time of diagnosis of CUA	Vitamin K antagonists/warfarin = 5 (15.2%)
	Non-vitamin K antagonist oral anticoagulant (NOAC) = 1 (3.0%)
	Iron supplements = 29 (87.9%)
	Erythropoiesis-stimulating agents (ESA) = 30 (90.9%)
	Calcium-based phosphate binders (CBPB) = 26 (78.8%)
	Non-CBPB = 13 (39.4%)
	Vitamin D = 26 (78.8%)
	Cinacalcet = 9 (27.3%)
	Statins = 19 (57.6%)
	Renin-angiotensin system blockers = 16 (48.5%)
	Beta blockers = 16 (48.5%)
	Insulin = 6 (18.2%)
Investigations	Calcium (mmol/L) = 2.44 ± 0.29
	Phosphate (mmol/L) = 2.18 ± 0.67
	CaXPO4 = 5.41 ± 1.90
	Albumin (g/L) = 27.73 ± 8.06
	Alkaline phosphatase (ALP) (U/L) = 364.47 ± 324.68
	Intact parathyroid hormone (iPTH) (pmol/L) = 181.14 ± 153.23
	Hemoglobin (g/dL) = 8.94 ± 1.84
	C-reactive protein (mg/L) = 128.43 ± 123.83
Diagnosis of CUA	Skin biopsy done = 18 (54.5%)
	Bone scan done = 3 (9.1%)
Distribution of lesions	Central = 10 (30.3%)
	Peripheral = 19 (57.6%)
	Both = 4 (12.1%)
	*Rare presentations-pulmonary calciphylaxis (1), heart and lung calcifications (1), liver and spleen calcifications (1), genital lesions (1)
Treatment of CUA	Use IV sodium thiosulphate = 10 (30.3%)
	Use non-CBPB = 19 (57.6%)
	Use cinacalcet = 16 (48.5%)
	Stop CBPB = 22 (66.7%)
	Parathyroidectomy = 11 (33.3%)
	Bisphosphonates = 2 (6.1%)
	Wound care = 31 (93.9%)
	Surgical debridement = 11 (33.3%)
	Increase dialysis frequency = 11 (33.3%)
	Switch PD to HD = 4 (12.1%)
	Intradialytic/total parenteral nutrition (IDPN/TPN) = 5 (15.2%)
Outcome of CUA	Mortality within 3 months = 18 (54.5%)
Time from diagnosis to death	4.12 ± 5.59 months
Cause of death	Sepsis/infection: 15 (45.5%)
	Cardiovascular disease: 1 (3.0%)
	Palliative: 2 (6.1%)
	Others: 5 (15.2%)
	Unknown: 10 (30.3%)

## Discussion

We reported the first and largest multicenter study on CUA in Malaysia, with an estimated incidence of 6.6 per 10,000 dialysis patients. Two-thirds of our cohort of CUA patients were on HD. A majority of the patients in Malaysia underwent HD as kidney replacement therapy; hence, our results appeared to correlate with this. In Malaysia, we have up to 49,434 patients who are on dialysis in 2020. Out of these, 88.7% are doing in-center HD, while the rest are undergoing PD [9]. In Malaysia, about 11% of our dialysis patients are on PD, and from our study up to one-third of CUA patients were on PD. This finding is consistent with data reported in other studies which showed a higher incidence of CUA among the PD population compared to that of HD [10]. The postulated reason for this is possibly a constant high level of phosphate in PD contributing to the pathology. This study postulated that contributing factors also support the therapeutic response of changing modality from PD to HD in CUA cases [11].

Worldwide data reported that the mean age of diagnosis of CUA is between 50 and 70 years [1]. Our cohort seemed to be younger with the mean age for diagnosis of CUA of 47.33 ± 13.80 years. A total of 69.7% of our CUA patients were female, and this is consistent with worldwide data which shows a predilection for women [1].

The Malaysia National Health and Morbidity Survey 2023 reported a prevalence of diabetes at 15.6%, and almost 84% of young adults aged 18-29 years are not aware that they have diabetes [12]. There is a similar trend of increasing prevalence in other noncommunicable diseases such as hypertension, high cholesterol, and obesity. Besides that, up to 84% of adult Malaysians are not active in sports [12]. The rise of diabetes is directly correlated with the rise of other chronic diseases such as chronic kidney disease. As a multiethnic upper-middle-income developing country in Southeast Asia, sedentary lifestyle and dietary habits, rapid urbanization, limited public awareness, and the lack of proper screening programs leads to the rising trend of noncommunicable diseases. When more patients start dialysis at younger age, dialysis vintage is longer, and this is a postulated possibility of why patients develop complications of ESKD such as CUA at a younger age.

Other risk factors for CUA as reported includes obesity, diabetes mellitus, and usage of VKAs or warfarin. From our study, only 18.2% of our patients are obese, and only 15.2% are on VKAs.

CUA is typically seen in patients receiving chronic dialysis for more than one year, and the mean dialysis vintage in our cohort is 6.2 years. Half of our CUA patients were diagnosed clinically, and not all patients underwent skin biopsy for confirmation. The possibility that some patients were not biopsied for histological confirmation could be due to concerns of poor wound healing and exacerbation of the ulcerations.

Management of CUA involves a multidisciplinary approach. Treatment is mainly targeted to address vascular calcifications, thrombosis, wound care, and pain control. The blood parameters of mineral bone disease in CUA patients are often deranged as evidenced in our patients. Only one-third of our patients had parathyroidectomy done. Possible reasons that other patients could not have parathyroidectomy were malnourished state or concomitant comorbid, making the patients unfit for operation. The role of sodium thiosulphate in CUA was first reported in 2004 [13]. The effectiveness of this medication is postulated due to its action as a chelating agent for calcium and iron and its antioxidative and vasodilatory properties. Sodium thiosulphate is not readily available in many hospitals, and only about one-third of patients were treated with this medication. The commonly prescribed dose is 25 g thrice weekly during HD.

Many have reported improvement in wound outcome with the use of hyperbaric oxygen therapy as an adjunctive treatment for CUA wound care and healing of tissues. Unfortunately, hyperbaric oxygen therapy is not widely accessible in Malaysia; hence, none of our patients had the opportunity to try this treatment.

Rheopheresis has been introduced as a potential promising approach for the treatment of CUA [14]. One of our CUA patients who had very resistant skin lesions despite more than six months of sodium thiosulphate treatment was given a trial of lipid apheresis for 10 sessions in one of the tertiary centers. There was subtle improvement with the lipid apheresis treatment whereby there were no new skin lesions developing after he was started on lipid apheresis. Further studies on this novel treatment approach will be needed.

Overall, there is still a lack of effective treatment strategies for CUA. Mortality rates for CUA remains high. Most studies worldwide reported a one-year mortality rate of 45%-80% [2,7-8]. The common cause of mortality in CUA is from infection of the ulcerated wounds leading to sepsis. Similar to other reported data, almost half of our patients died due to infection.

It is difficult to perform randomized studies for CUA due to the high mortality rates and low incidence of disease, especially in a small country like Malaysia. Several countries like Germany, USA, and the UK have established international CUA registries to increase the awareness and to promote knowledge of this entity.

## Conclusions

This is the first and largest multicenter study looking into the characteristics, treatment, and outcome of CUA in Malaysia. Majority of patients in Malaysia undergo HD as kidney replacement therapy; hence, our results correlate with this. The incidence of CUA was estimated to be 6.6 per 10,000 dialysis patients in this study, and the mortality rate is very high. This is consistent with worldwide data which reported mortality as high as 60%. In conclusion, CUA is not an uncommon condition, and probably many cases are underreported. There is a need to increase awareness among clinicians in Malaysia about this condition and its risk factors. In the long run, as clinicians gain more knowledge on this condition, it is hope that patient’s mineral bone disorders are identified early and managed well to reduce the incidence, morbidity, and mortality from CUA. 
